# Preeclampsia-Like Features and Partial Lactation Failure in Mice Lacking Cystathionine γ-Lyase—An Animal Model of Cystathioninuria

**DOI:** 10.3390/ijms20143507

**Published:** 2019-07-17

**Authors:** Noriyuki Akahoshi, Hiroki Handa, Rintaro Takemoto, Shotaro Kamata, Masahide Yoshida, Tatsushi Onaka, Isao Ishii

**Affiliations:** 1 Department of Health Chemistry, Showa Pharmaceutical University, Tokyo 194-8543, Japan; 2 Department of Physiology, Jichi Medical University, Tochigi 329-0498, Japan

**Keywords:** cystathionine γ-lyase, homocysteine, hypertension, milk ejection, oxytocin, preeclampsia, proteinuria

## Abstract

Elevated plasma homocysteine levels are considered as a risk factor for cardiovascular diseases as well as preeclampsia—a pregnancy disorder characterized by hypertension and proteinuria. We previously generated mice lacking cystathionine γ-lyase (Cth) as cystathioninuria models and found them to be with cystathioninemia/homocysteinemia. We investigated whether Cth-deficient (*Cth*^−/−^) pregnant mice display any features of preeclampsia. *Cth*^−/−^ females developed normally but showed mild hypertension (~10 mmHg systolic blood pressure elevation) in late pregnancy and mild proteinuria throughout development/pregnancy. *Cth*^−/−^ dams had normal numbers of pups and exhibited normal maternal behavior except slightly lower breastfeeding activity. However, half of them could not raise their pups owing to defective lactation; they could produce/store the first milk in their mammary glands but not often provide milk to their pups after the first ejection. The serum oxytocin levels and oxytocin receptor expression in the mammary glands were comparable between wild-type and *Cth*^−/−^ dams, but the contraction responses of mammary gland myoepithelial cells to oxytocin were significantly lower in *Cth*^−/−^ dams. The contraction responses to oxytocin were lower in uteruses isolated from *Cth*^−/−^ mice. Our results suggest that elevated homocysteine or other unknown factors in preeclampsia-like *Cth*^−/−^ dams interfere with oxytocin that regulates milk ejection reflex.

## 1. Introduction

Elevated plasma levels of homocysteine, a sulfur-containing amino acid intermediate in methionine metabolism, are widely known as an independent risk factor for cardiovascular diseases (CVDs) such as myocardial infarction, stroke, and venous thromboembolism [1,2,3,4,5,6]. In addition, an increase in the plasma homocysteine levels during pregnancy is attributed to pregnancy complications including preeclampsia, (recurrent) spontaneous abortion, premature/low-body-weight infants [7,8,9,10], and neonatal neural tube defects (NTDs) such as spina bifida, hydrocephaly, and anencephaly/iniencephaly [11]. Therefore, folic acid food/supplement fortification is underway in many countries to lower plasma homocysteine levels (by activating remethylation of homocysteine to methionine) and thereby prevent the onset/progression of CVDs, pregnancy complications, and NTDs [12]. The beneficial impacts of folic acid fortification on pregnancy complications and NTDs have been rather epidemiologically approved [13] but those on CVDs continue to be a matter of controversy [14,15,16].

Homocysteinemia could be caused by several genetic factors, including deficiencies of cystathionine β-synthase (CBS) (homocystinuria; MIM 236200), 5,10-methylenetetrahydrofolate reductase (MIM 236250), or methionine synthase (MIM 250940) [6]. Patients with CBS-deficient “classical” homocystinuria show a variety of clinical symptoms including atherothrombosis, mental retardation, osteoporosis, lens dislocation, and Marfan-like skeletal abnormality [6]. Therefore, for these reasons, homocystinuria is listed among the newborn screening tests in most developed countries [17]. Cbs-deficient mice have been generated as an animal model of homocystinuria, and they showed homocysteinemia/methioninemia and hepatic steatosis; severe juvenile deaths with unknown reasons (not NTDs) have been reported in these mice [18,19,20]. Since rare homozygous *Cbs*^−/−^ survivors (both females and males) were found to be infertile (our unpublished observation), pregnancy complications could not be investigated. In our previous study, we generated mice lacking cystathionine γ-lyase (Cth), which is another essential transsulfuration enzyme downstream of Cbs, as a model for cystathioninuria (MIM 219500) [21]. Hereditary cystathioninuria/cystathioninemia due to CTH deficiency has been considered as a benign biochemical anomaly without any striking clinical/pathophysiological manifestations [6,22]. Our homozygous *Cth*^−/−^ mice appeared normal and showed cystathioninemia/uria expectedly, but also homocysteinemia/homocystinuria unexpectedly [21,23]. Therefore, we first aimed to reveal whether homocysteinemic *Cth*^−/−^ dams display any features of preeclampsia such as hypertension and proteinuria.

Previous studies have also shown that the Cth expression was upregulated in the livers of lactating mouse [24] or rat [25] dams, and that the pharmacological inhibition of Cth by propargylglycine (PPG) in rat dams significantly inhibited daily weight gain of their pups, which was canceled by *N*-acetylcysteine administration [25]. Moreover, PPG inhibition of Cth in lactating rats mimicked the changes in the gene and protein expression profiles of mammary glands in weaned rats [26] while Cth inhibition (by PPG) in pregnant mice reduced plasma hydrogen sulfide (H_2_S) levels and increased the blood pressure/irregular branching of the fetal vasculature of the placenta, both of which were reversed by the application of GYY4137, a slow-releasing H_2_S donor [27]. Therefore, we secondly aimed to investigate the maternal behavior in *Cth*^−/−^ dams. In this study, we report preeclampsia-like hypertension/proteinuria during late pregnancy as well as partial lactation (milk ejection) failure due to impaired oxytocin responses in *Cth*^−/−^ mice. Our results shed light on the novel, clinically relevant roles of Cth and homocysteine in the systemic circulation.

## 2. Results

### 2.1. Hypertension and Proteinuria During Late Gestation Periods in Cth^−/−^ Dams

**Figure 1:**
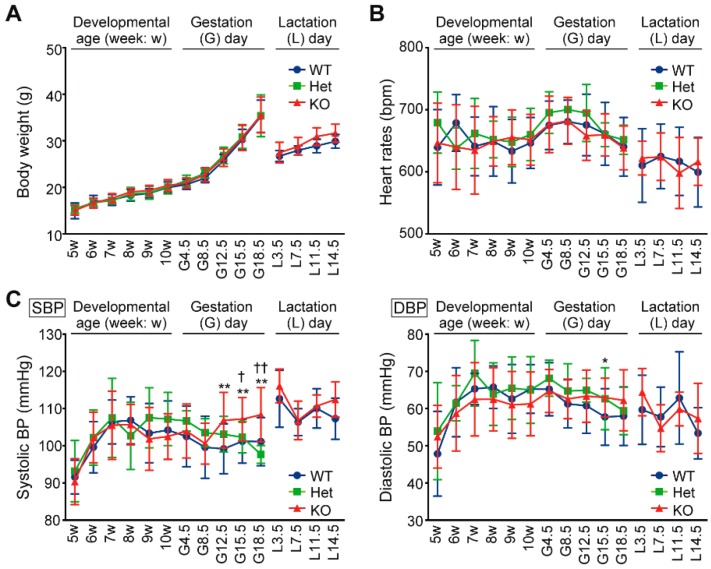
Pregnancy-induced hypertension in *Cth*^−/−^ dams. Changes in body weights (**A**), heart rates (**B**), and systolic/diastolic blood pressures (SBP/DBP) (**C**) of wild-type (*Cth*^+/+^, WT, *blue*), heterozygous (*Cth*^+/−^, Het, *green*), and homozygous (*Cth*^−/−^, KO, *red*) female mice during juvenile development (5–10 week), gestation days (G4.5–G18.5), and lactation days (L3.5–L14.5). Blood pressures and heart rates were measured using a tail-cuff manometer. Data are represented as mean ± SD (*n* = 9–18 for juvenile development, 21–22 for gestations days, and 5 for lactation days in *Cth*^+/+^; *n* = 7–12 for juvenile development and 5–6 for gestation days in *Cth*^+/−^; and *n* = 9–20 for juvenile development, 15–16 for gestation days, and 6 for lactation days in *Cth*^−/−^). The differences between *Cth*^+/+^ and *Cth*^−/−^ were significant at * *p* < 0.05 and ** *p* < 0.01 and those between *Cth*^+/−^ and *Cth*^−/−^ were significant at ^†^ *p* < 0.05 and ^††^ *p* < 0.01 in Student’s *t*-test.

**Figure 2:**
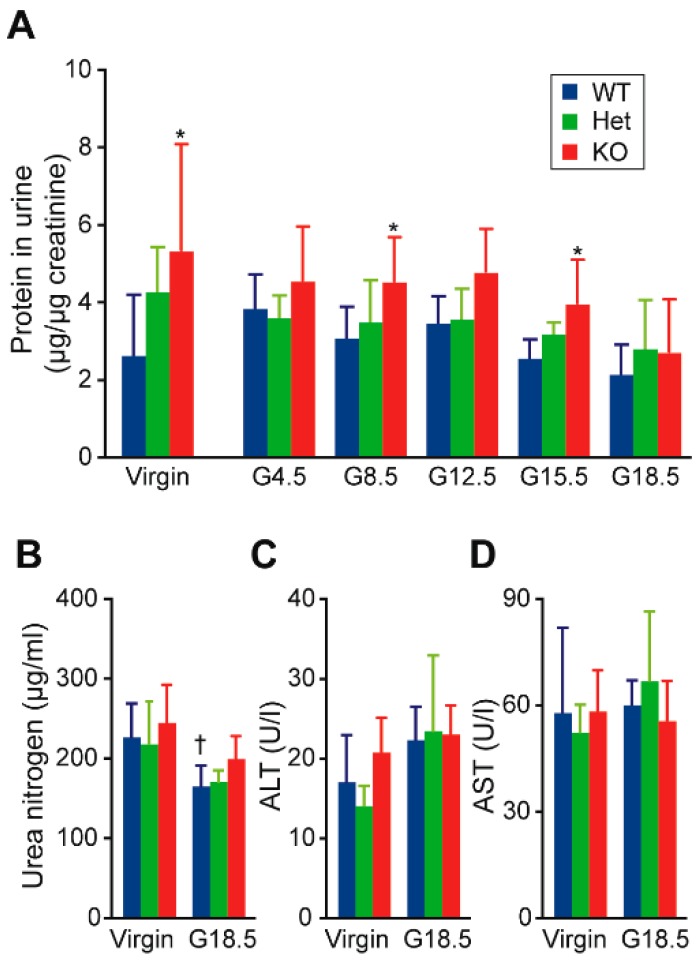
Proteinuria in *Cth*^−/−^ female mice. (**A**) Urinary protein concentrations of wild-type (WT), *Cth*^+/−^ (Het), and *Cth*^−/−^ (KO) female mice were measured at 8 weeks of age (virgin) and gestating (G) days (G4.5, G8.5, G12.5, G15.5 and G18.5), and normalized by their blood/urine creatinine levels. Urinary samples were collected as temporary urine at 8 weeks of age (virgin) and at G4.5–G18.5. (**B**–**D**) Serum levels of urea nitrogen (**B**), alanine aminotransferase (ALT) (**C**), and aspartate aminotransferase (AST) (**D**) were measured. Data are represented as mean ± SD (*n* = 4–18). The differences *versus* WT samples were significant at * *p* < 0.05 and those versus virgin samples were significant at ^†^ *p* < 0.05.

Both heterozygous *Cth*^+/−^ and homozygous *Cth*^−/−^ female mice grew in a similar manner as wild-type (WT: *Cth*^+/+^) females during juvenile development, gestation, and lactation periods (Figure 1A) while maintaining normal heart rates (Figure 1B). Although *Cth*^−/−^ female mice were normotensive during juvenile development (Figure 1C *left* and [21,28]), their systolic blood pressures during gestation day 12.5 (G12.5), G15.5, and G18.5 were ~10 mmHg higher (*p* < 0.01) than those of the respective WT females (Figure 1C *left*). Systolic blood pressures during lactation periods and diastolic blood pressures in all periods (except G15.5 in *Cth*^−/−^; *p* < 0.05) were indistinguishable between the *Cth* genotypes (Figure 1C *left* and *right*, respectively). Urinary protein levels (normalized by urinary creatinine) were significantly higher (*p* < 0.05) in virgin *Cth*^−/−^ mice than those in the respective WT mice, which lasted to G15.5 (Figure 2A). Urinary protein levels were intermediate in *Cth*^+/−^ mice (Figure 2A). Serum levels of urea nitrogen, alanine aminotransferase (ALT), and aspartate aminotransferase (AST) were all comparable between the *Cth* genotypes in both virgin and G18.5 dams, suggesting their generally normal renal/hepatic functions (Figure 2B–D, respectively).

### 2.2. Frequent Neonatal Deaths in Cth^+/−^ Pups Born to Cth^−/−^ Dams

**Figure 3:**
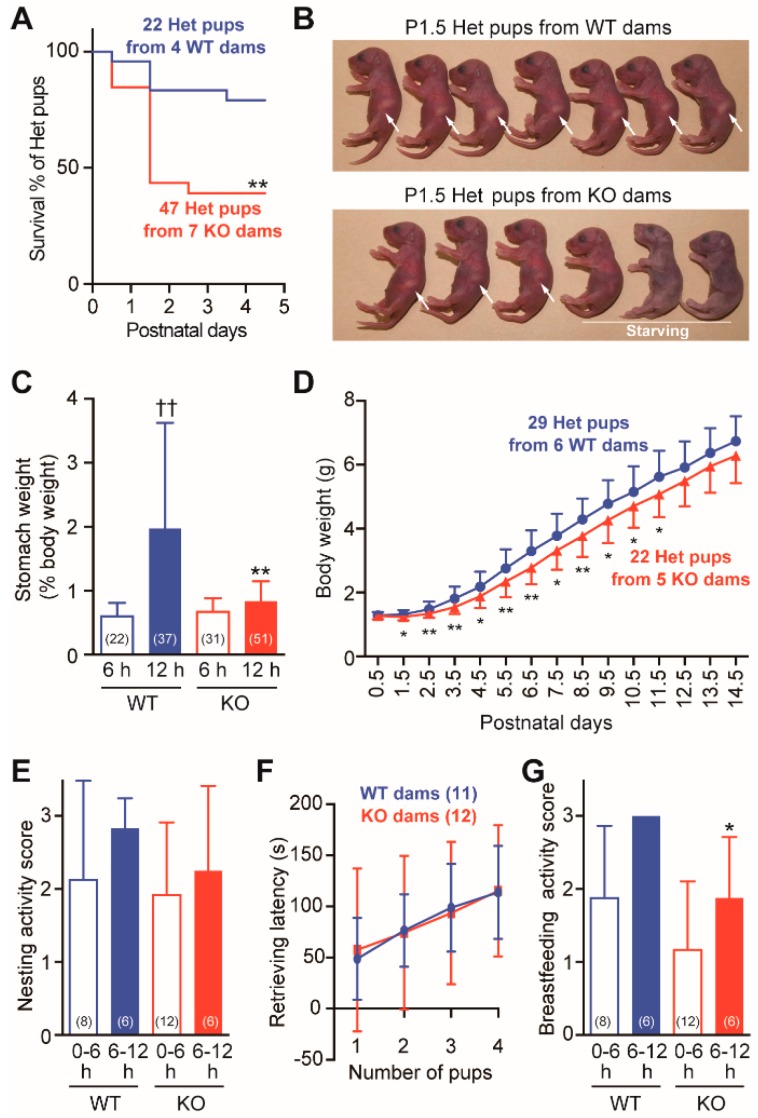
Frequent neonatal lethality in pups born to *Cth*^−/−^ dams due to defective lactation. (**A**) Kaplan–Meier survival analyses up to postnatal day 5 in *Cth*^+/−^ pups born to wild-type (WT) and *Cth*^−/−^ (KO) dams. The difference was significant at ** *p* < 0.01. (**B**) Typical appearance of all surviving *Cth*^+/−^ litters from a WT dam and partially dying *Cth*^+/−^ litters from a KO dam. Arrows indicate the presence of milk in their stomachs. (**C**) Gastric weight (with milk) percentages of body weights (at 6 or 12 h after birth) of *Cth*^+/−^ pups born to WT and KO dams. Data are represented as mean ± SD (*n* in parentheses) and the differences versus 6 h samples of the same genotype were significant at ^††^ *p* < 0.01 and those versus WT samples at the same postnatal periods at ** *p* < 0.01 in Student’s *t*-test. (**D**) Bodyweight changes up to P14.5 in (surviving) *Cth*^+/−^ pups born to WT or KO dams. The differences were significant at * *p* < 0.05 and ** *p* < 0.01 in Student’s *t*-test. (**E**) Nesting activity was scored (0, 1, 2, or 3) during 0–6 and 6–12 postnatal hours. No significant difference was found by Mann–Whitney *U* test. (**F**) Latency (in s) to retrieve 1–4 pups to their nest was counted when five pups were placed in the diagonal position of the cage. No significant difference was found by Student’s *t* test. (**G**) Breastfeeding activity was scored (0, 1, 2, or 3) during 0–6 and 6–12 postnatal hours. The difference versus WT mice was significant at * *p* < 0.05 in the Mann–Whitney *U* test.

We noticed in routine breeding that neonatal death rates (by postnatal day 5 [P5]) in *Cth*^+/−^ pups born to *Cth*^−/−^ dams (resulting from their mating with WT males) were significantly higher (*p* < 0.01) than that in *Cth*^+/−^ pups born to WT dams (resulting from their mating with *Cth*^−/−^ males) (Figure 3A). Compared to P1.5 *Cth*^+/−^ pups born to WT dams, those born to *Cth*^−/−^ dams lacked intragastric milk; therefore, they often died of hunger (Figure 3B). Although the gastric weights (with milk) were indistinguishable between WT and *Cth*^−/−^ dams at 6 h after birth, those of WT dams were more than twice as high as those of *Cth*^−/−^ dams at 12 h after birth (*p* < 0.01; Figure 3C). It is notable that all the *Cth*^+/−^ littermates were lost (dead) in nearly half of the litters of *Cth*^−/−^ dams while not all the *Cth*^+/−^ littermates in any of the litters of WT dams died (data not shown). In the remaining half of the litters born to Cth^−/−^ dams, all the Cth^+/−^ pups survived to adulthood even though they showed slightly retarded growth in early development (*p* < 0.05 or 0.01 during P1.5–P11.5; Figure 3D). When maternal behaviors were analyzed during the lactation periods, the nesting activity scores (that evaluate maternal care of pups by crouching over them; Figure 3E) and the latencies to retrieve 1–4 pups when five pups were placed outside of the nest were comparable between WT and Cth^−/−^ dams during both 0–6 and 6–12 postnatal hours (Figure 3F). In contrast, the breastfeeding activity scores (that evaluate lactating behaviors) were significantly lower in Cth^−/−^ dams only at 6–12 h (*p* < 0.05; Figure 3G). These results indicate that Cth^−/−^ dams were occasionally unable or had some difficulty in breastfeeding their pups in efficient/appropriate manners.

### 2.3. Defective Milk Ejection from Cth^−/−^ Dams

**Figure 4:**
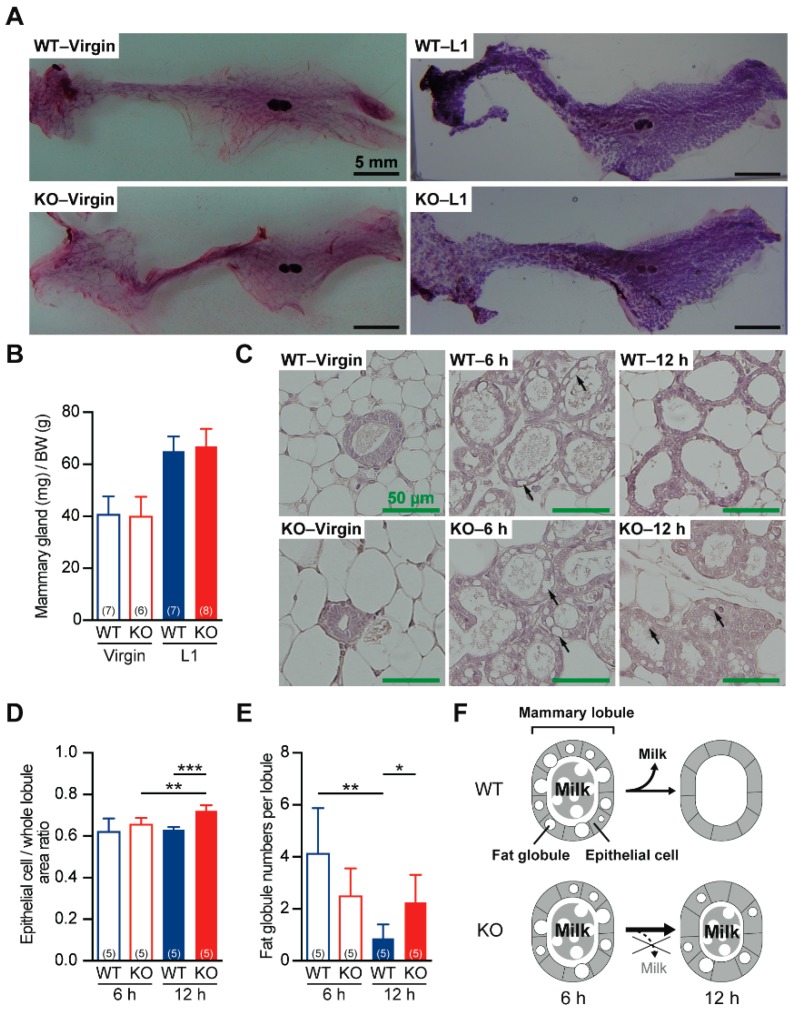
Histological analysis of the mammary glands from virgin and lactating mice. (**A**) Carmine alum staining of whole mammary glands isolated from virgin/lactating day 1 (L1) WT and *Cth*^−/−^ (KO) mice. Bars are 5 mm. (**B**) Mammary gland weight changes normalized by body weights. Data are represented as mean ± SD (*n* in parentheses). (**C**) Hematoxylin/eosin-stained mammary gland sections from virgin, 6 h-postpartum, and 12 h-postpartum WT and KO mice. Bars are 50 µm. (**D**) Epithelial cell/whole globule area ratios upon 6 and 12 h lactation. The area ratios of 12 h-postpartum KO mice were higher than those of 6 h-postpartum KO mice and 12 h-postpartum WT mice at ** *p* < 0.01 and *** *p* < 0.001, respectively. (**E**) Fat globule numbers per mammary globule. The numbers became smaller at 12 h after birth in WT mice but not in KO mice. The differences were significant at * *p* < 0.05 and ** *p* < 0.01. (**F**) Schematic illustration of the changes in the mammary gland sections upon lactation in WT and KO mice.

The postpartum development of the mammary glands in *Cth*^−/−^ dams was equivalent to that of the mammary glands in WT dams (Figure 4A,B), suggesting normal actions of prolactin, a luteotropic hormone that regulates mammary gland development and milk production [29], in *Cth*^−/−^ dams. Histological analyses revealed normal architectures of the mammary lobules in virgin and 6 h-postpartum *Cth*^−/−^ dams (Figure 4C); however, epithelial cell/whole globule area ratios became significantly higher (i.e., epithelial cell hypertrophy/thickening) in 12 h-postpartum *Cth*^−/−^ dams (*p* < 0.001; Figure 4C,D). Furthermore, the milk maintained in the mammary lobules of 6 h-postpartum WT dams were mostly consumed by ejection during the next 6 h, whereas the milk of 6 h-postpartum *Cth*^−/−^ dams substantially remained in the lobules along with the fat globules (Figure 4C,E). These results suggest that *Cth*^−/−^ dams showed defective ejection of the first milk, as schematically illustrated in Figure 4F.

### 2.4. Impaired Milk Ejection Responses to Oxytocin in Cth^−/−^ Dams

**Figure 5:**
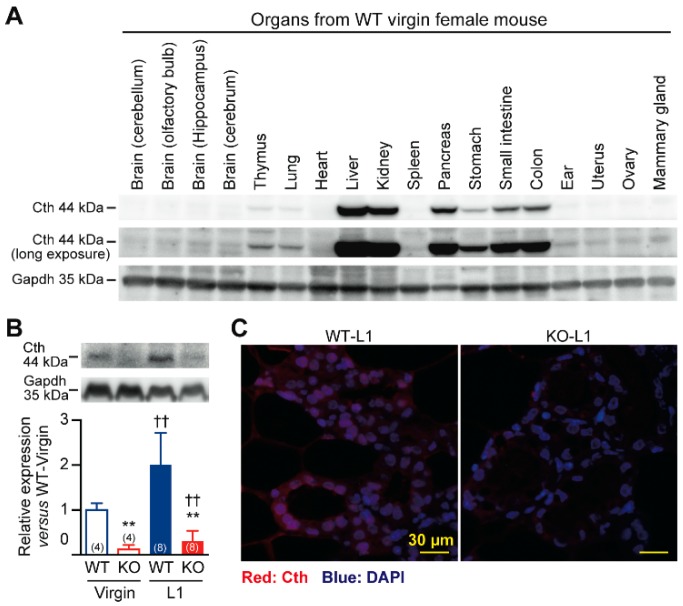
Cth expression in the mammary gland. (**A**) Western blot analyses of Cth expression in various tissues of wild-type (WT) virgin female mice. The 44 kDa Cth proteins (short and long exposures) and 35 kDa Gapdh proteins (as controls) are shown. (**B**) Cth expression in the mammary glands of the virgin/lactating day 1 (L1) WT and *Cth*^−/−^ (KO) mice. The 44 kDa bands in KO tissues are non-specific. Data are mean ± SD (*n* in parentheses) and significant differences were observed versus WT at ** *p* < 0.01 and versus virgin at ^††^ *p* < 0.01. (**C**) Cth immunostaining (*red*) of mammary gland sections. The nuclei were stained with DAPI (blue). Bars are 30 µm.

In virgin WT female mice, Cth was mainly expressed in the liver, kidney, pancreas, and smooth muscle organs including the stomach, small intestine, and colon, although it was also substantially expressed in the thymus and lung, followed by ear, uterus, ovary, and mammary glands (Figure 5A). Cth expression in the mammary glands of WT mice was upregulated at L1 (*p* < 0.01; Figure 5B), suggesting its physiological roles in milk production/ejection. Although some background signals and their upregulation upon lactation were observed in the mammary glands of *Cth*^−/−^ mice (long exposed Cth images in Figure 5B), the complete absence of *Cth* gene expression was confirmed by RT-PCR (data not shown). Histological analyses localized Cth expression in the myoepithelial cells of WT mouse mammary glands, but this finding was absent or unclear in those of *Cth*^−/−^ mouse mammary glands (Figure 5C).

**Figure 6:**
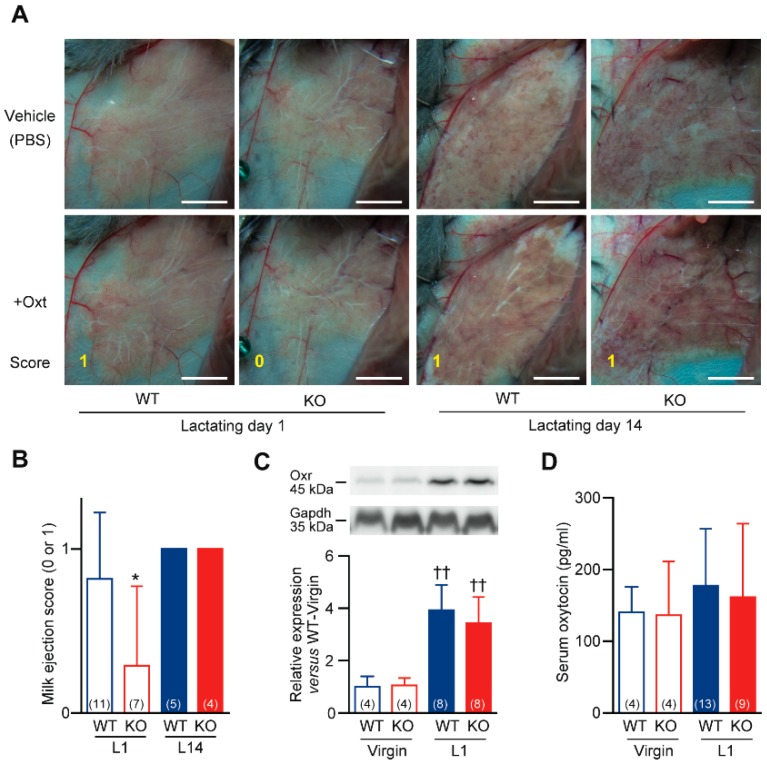
Impaired oxytocin-induced milk ejection in the epithelial ducts of *Cth*^−/−^ mammary gland. (**A**) Abdominal regions of lactating day 1 (L1)/L14 wild-type (WT) and *Cth*^−/−^ (KO) mice were exposed and oxytocin (0.04 unit) was applied. The increased amount of milk in the larger duct was detected (score 1) or not detected (score 0). (**B**) Milk ejection scores in L1/L14 WT and KO mice. Milk ejection scores were the smallest in L1 KO mice (* *p* < 0.05 versus L1 WT and L1/L14 KO). (**C**) Western blot analyses of oxytocin receptor (Oxr) in virgin/L1 WT and KO mice. Oxr expression was upregulated in L1 WT and KO mice (^††^ *p* < 0.01 versus their respective virgin) to similar levels. (**D**) Serum oxytocin levels in virgin/L1 WT and KO mice. Data are represented as mean ± SD (*n* in parentheses).

When an infant stimulates the nerve endings of the nipple through sucking, the sensory impulses travel through the spinal cord to the synchronization center in the hypothalamus. Then, the activated supraoptic and paraventricular nucleus release oxytocin to the systemic circulation. Mammary gland myoepithelial cells respond to a peptide hormone oxytocin by contracting and forcing milk from the alveolar space into the ducts as called milk ejection reflex [30,31,32]. The myoepithelial cell contraction responses to oxytocin (0.04 unit) were detected after 1 min as an increased amount of milk in the larger ducts (score 1) in both isoflurane-anaesthetized-laparotomized WT and *Cth*^−/−^ dams at L14 (Figure 6A,B). In contrast, the same oxytocin administration often failed to induce any apparent change (score 0) only in *Cth*^−/−^ dams at L1 (Figure 6A,B). In the mammary gland, only the myoepithelial cells express the G protein-coupled oxytocin receptor (Oxtr) [33], and the impaired responses in *Cth*^−/−^ dams could be attributed to decreased Oxtr expression in their mammary glands. Although the Oxtr expression in the mammary glands was highly enhanced by lactation (*p* < 0.01), its levels were indistinguishable between WT and *Cth*^−/−^ dams (Figure 6C). Serum levels of oxytocin showed a trend toward increase upon lactation (although not statistically significant), but there were no differences in the serum oxytocin levels between WT and *Cth*^−/−^ dams (Figure 6D).

### 2.5. Impaired Contraction Responses to Oxytocin in Cth^−/−^ Dam Uteruses

**Figure 7:**
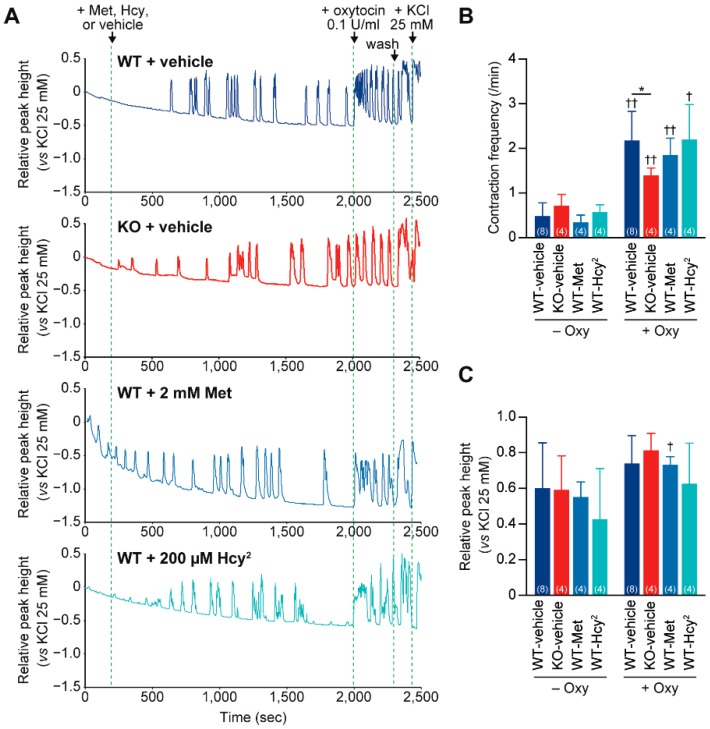
Impaired oxytocin-induced uterine contraction in *Cth*^−/−^ mice. The uteruses were isolated from anaesthetized wild-type (WT) and *Cth*^−/−^ (KO) mice and their contraction responses to oxytocin (Oxy; final 0.1 unit/mL) were evaluated using the Magnus apparatus. (**A**) The representative amplified electric signals for contraction-relaxation reactions of WT and KO uteruses, and those of WT uteruses in the incubation buffer containing 2 mM methionine (Met) or 200 µM homocystine (Hcy^2^). (**B**) Contraction (/relaxation) frequency per minute. Data are represented as mean ± SD (*n* in parentheses). The differences versus WT-vehicle samples were significant at * *p* < 0.05 and those between −Oxy and +Oxy samples were significant at ^†^ *p* < 0.05 and ^††^ *p* < 0.01. (**C**) The relative contraction peak height compared to that in KCl 25 mM. The differences between −Oxy and +Oxy samples were significant at ^†^ *p* < 0.05.

Cth was expressed in the uterus (Figure 5A) and oxytocin is known to drastically increase the strength and frequency of uterine contraction [32]. Therefore, we next investigated the oxytocin-induced contraction of WT and *Cth*^−/−^ mouse uteruses by using the conventional Magnus apparatus. After some spontaneous contractions (with a low frequency), WT uteruses markedly responded to oxytocin (0.1 unit/mL) with increased frequency (×4.50) and higher peak values (×1.23; though not statistically significant) (Figure 7A–C). In contrast, the responses of *Cth*^−/−^ uteruses were much milder (×1.95) in contraction frequency (Figure 7A–C). As *Cth*^−/−^ mice show homocysteinemia [21], we next examined the effects of methionine or homocystine (a homocysteine dimer) application to the chamber in order to elevate the intracellular homocysteine levels in WT uteruses so as to somehow mimic the *Cth*^−/−^ uterus. Although the application of high concentrations of methionine (2 mM) failed to affect both the contraction frequency and magnitude, homocystine (200 µM) showed a trend toward lower contraction magnitudes in both spontaneous and oxytocin-induced conditions (though not statistically significant) without altering the contraction frequency (Figure 7A–C).

If the systemic oxytocin signaling is impaired in *Cth*^−/−^ female mice, their responses to vasopressin, another peptide hormone that resembles oxytocin (only two amino acid substitutions in a nine amino acid peptide) and that acts on the Oxtr-ortholog receptor, vasopressin receptor 2 (V2r) [34], could also be interfered. Vasopressin increases the renal reabsorption of solute-free water back into the circulation and V2r-deficient mice showed increased water intake and increased urine output (Diabetes insipidus) [35]; however, daily (24 h) urine amounts and water intakes were indistinguishable between virgin adult WT and *Cth*^−/−^ female mice (5.38 ± 1.12 and 4.83 ± 0.67 g per 20 g body weight for urine amounts; 6.48 ± 1.81 and 5.77 ± 0.89 g per 20 g body weight for water intake, respectively).

### 2.6. Normal Social Memory/Recognition in Cth^−/−^ Male Mice

**Figure 8:**
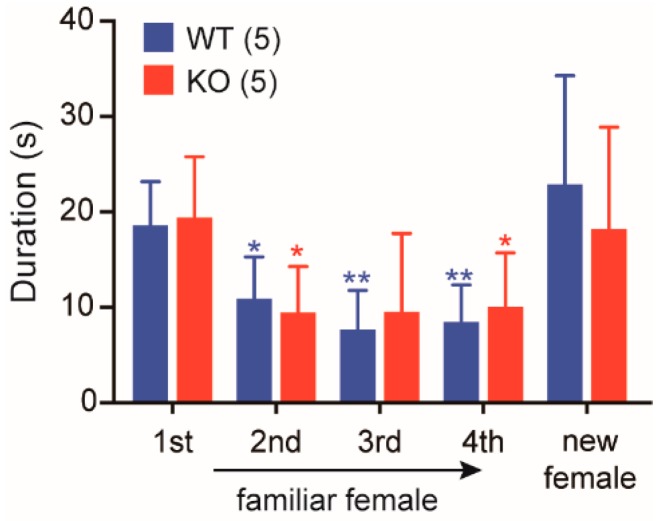
Normal social memory/recognition in *Cth*^−/−^ male mice. Social memory/recognition by wild-type (WT) and *Cth*^−/−^ male mice was evaluated by duration time (s) spent for anogenital investigation of unfamiliar females during 1st to 4th 1 min-trials with 10-min intervals. A 5th “dishabituation” trial depicts the response of males to the presentation of a new female. Data are represented as mean ± SD (*n* in parentheses) and the differences versus 1st trial were significant in * *p* < 0.05 and ** *p* < 0.01.

The above investigations demonstrated rather normal maternal behaviors (only except slightly low nursing activity) that can be regulated by oxytocin within the central nervous system (CNS) as well as substantially impaired peripheral oxytocin-mediated responses, milk ejection and uterine contraction, in *Cth*^−/−^ dams. To evaluate another oxytocin-mediated signaling in the CNS [36], social memory/recognition tests were applied to WT and *Cth*^−/−^ male mice. When a male mouse is exposed to an “unfamiliar” female in his home cage, he spends much of his time in the anogenital inspection of the novel individual during the brief social encounter. Upon the repetitive exposures to the “familiar” female, the time spent for investigation usually declines with a full recovery following the introduction of a new female [36,37,38]. WT and *Cth*^−/−^ male mice displayed declines in the investigation time upon the repetitive exposures to the “familiar” females (×0.58 and ×0.49 at the 2nd exposure; ×0.45 and ×0.46 at the 3rd exposure; ×0.44 and ×0.53 at the 4th exposure, respectively), and the recoveries of the time upon new females in a similar fashion (×1.31 and ×0.95, respectively; Figure 8), indicating the normal social memory/recognition activity in *Cth*^−/−^ males.

### 2.7. Cth^−/−^ Dam Milk With Altered Amino Acid Profiles Did not Affect Serum Amino Acid Profiles in Their Pups

*Cth*^−/−^ virgin adult female mice exhibited serum amino acid profiles that were different from those of the respective WT mice as we previously reported in 2-week-old female/male-mixed pups (e.g., high Arg, Met, cystathionine, citrulline, and total homocysteine levels; Table 1) [23]. Serum amino acid levels of WT primiparas that were nursing 2-week-old pups differed from those of WT virgin females; the WT primiparas showed lower Arg, Tyr, and citrulline levels and higher Glu, Met, taurine, total Cys, total GSH, total Cys-Gly, and total γ-Glu-Gly levels (Cys-Gly and γ-Glu-Gly are precursors/metabolites of GSH) (Table 1). However, the differences between the virgins and primiparas were much more pronounced in *Cth*^−/−^ mice, which had much higher Gln, His, Ile, Leu, Lys, Val, cystathionine, citrulline, ornithine, and total homocysteine levels. Some alterations were also reflected in the whey amino acid profiles of *Cth*^−/−^ primiparas (Table 1). Even such drastic changes in whey could not be attributed to the delayed growth in *Cth*^+/−^ pups born to *Cth*^−/−^ dams (Figure 3D) because the serum amino acid profiles in 2-week-old *Cth*^+/−^ pups born to *Cth*^−/−^ dams were comparable to those in respective *Cth*^+/−^ pups born to WT dams (Table 2).

## 3. Discussion

Cth is a transsulfuration enzyme essential for homocysteine clearance, cysteine biosynthesis, endogenous production of the gasotransmitter H_2_S, and protection against oxidative injuries and environmental electrophiles, as evidenced by our previous studies demonstrating that (1) *Cth*^−/−^ mice display (hyper)homocysteinemia/cystathioninemia [21]; (2) *Cth*^−/−^ mice require dietary cyst(e)ine as an essential amino acid (unlike WT mice) [21]; (3) *Cth*^−/−^ mice show reduced endogenous H_2_S levels [39,40,41], and (4) *Cth*^−/−^ mice display increased vulnerability to paraquat [21], dietary Met [42], acetaminophen [43,44], cardiac ischemia/reperfusion injury [28], unilateral ureteral obstruction-induced kidney fibrosis [40], cadmium [45,46], and methyl mercury [46,47]. However, *Cth*^−/−^ mice appeared normal under normal condition and were fertile [21]. Patients with cystathioninuria have been rarely found to be free of the apparent clinical symptoms [6,22]. Therefore, patients with silent CTH-deficient cystathioninuria that exist with a low but substantial frequency should be carefully examined; CBS deficiency had been detected with a frequency between 1:200,000 and 1:335,000 [6].

Our *Cth*^−/−^ dams showed hypertension during pregnancy and mild proteinuria (perhaps associated with renal failure) during development/pregnancy (Figure 1 and Figure 2), which are the typical features of pregnancy hypertension syndrome/preeclampsia [48], but we could not clarify whether these preeclampsia-like phenotypes were induced by homocysteinemia. Because *Cth*^−/−^ pregnant mice performed normally in the parturition (data not shown) and in the initial nursing (Figure 3E), accidental neonatal deaths due to rare hypothermia by neglect outside the nest or puppy abuse occurred to a similar extent in WT and *Cth*^−/−^ dams within 1 day after birth (Figure 3A). If maternal care except lactation is normal, mouse neonates can live without milk over 1 day by autophagic degradation of “self” proteins [49]. Half of the *Cth*^+/−^ pups born to *Cth*^−/−^ dams were found dead between 1 and 2 postnatal days (Figure 3A,B), suggesting that there were some problems in the initial lactation. We found the deficits in oxytocin-induced milk ejection (Figure 4 and Figure 6) but not in the general maternal behavior (Figure 3E,F) except slightly lower breastfeeding activity (Figure 3G) in *Cth*^−/−^ dams. *Cth*^−/−^ male mice also showed normal social memory/recognition activity (Figure 8) that was absent in mice lacking the oxytocin gene (*Oxt*^−/−^) [38]. We did not observe the premature parturition in *Cth*^−/−^ and heterozygous *Cbs*^+/−^ (both homocysteinemic; data not shown) dams, as previously reported in *Cbs*^+/−^ dams by Sonne et al. [50]. In addition, we did not observe abnormal serum amino acid profiles in pups born to *Cth*^−/−^ dams (Table 2) that could cause neonatal deaths.

Responses or behaviors displayed by the female that specifically support the development and growth of her pups constitute a set of responses termed as maternal behavior. These include (1) parturitional responses such as neonate stimulation, amniotic fluid consumption, and placentophagia; (2) offspring-directed responses such as retrieval, licking/grooming, nursing/crouching (warming), and nest building; and (3) offspring-related responses, including maternal aggression, increased food consumption, and reduced anxiety to enhance exploratory activities [51]. Among the various neuroendocrine factors/hormones including estrogen, progesterone, prolactin, vasopressin, and classical neurotransmitters (e.g., dopamine, norephinephrine, serotonin) that are known to regulate maternal behavior, the fundamental roles of the neuropeptide oxytocin and its receptor Oxtr in maternal behavior, especially the offspring-directed responses, have been elucidated [32,52]. Oxytocin is synthesized in the magnocellular neurons of the supraoptic and paraventricular nuclei of the hypothalamus and then released to the peripheral circulation from the posterior pituitary [53]. Oxytocin is most often associated with milk ejection and uterine contraction [32,53]; meanwhile, lesser amounts of oxytocin are produced by paraventricular and other forebrain nuclei and released into the CNS to regulate social/maternal behaviors [32,53].

Oxytocin acts on the G_q_-coupled Oxtr and thereby activates phospholipase C and intracellular calcium signaling, which triggers various downstream signaling pathways. Previous studies using *Oxt*^−/−^ and mice lacking Oxtr (*Oxtr*^−/−^) revealed that both *Oxt*^−/−^ and *Oxtr*^−/−^ females have normal fertility, pregnancy, and parturition, but they were unable to eject milk; therefore, all pups born to these mice die within 24 h of birth due to the deficiency of milk [53,54,55]. In contrast, nearly half of the litters born to *Cth*^−/−^ dams could survive (Figure 3A) and therefore, oxytocin signaling seems to partially function in *Cth*^−/−^ dams. The slightly lower breastfeeding activity at 6–12 h after delivery (Figure 3G) could reflect the existence of pups that start to starve under the arms or the bellies of their dams. Taken together, we conclude that partial milk ejection failure due to defective oxytocin responses is attributed to occasional juvenile deaths in pups born to *Cth*^−/−^ dams.

What makes the peripheral (rather than the CNS) oxytocin signaling less active in *Cth*^−/−^ dams? One plausible explanation is homocysteinemia in *Cth*^−/−^ dams. Indeed, the application of homocystine (not Met at a higher dose) reduced uterine contraction magnitudes (though not significant; Figure 7C). In human and mouse blood, perhaps 75% of the total homocysteine (tHcy) is bound to proteins through disulfide bonds with protein cysteines, mainly in the albumin, and the majority of the remaining are homocysteine or homocystine-cysteine dimers [23,56]. The nine amino acid peptide oxytocin has an intermolecular disulfide bond between its two Cys residues and elevated blood levels of homocystine (or homocysteine-cysteine) might disrupt the disulfide bond via a new disulfide linkage and interrupt oxytocin signaling. However, this is unlikely because the V2r-mediated signaling of a similar peptide vasopressin was intact (normal urine amounts; Result 2.5.). Instead, elevated levels of homocyst(e)ine could somehow interfere with oxytocin signaling within the cells (e.g., mammary gland myoepithelial cells). Interestingly, we recently found that the total homocysteine levels of the cerebrospinal fluid were much lower than those of the serum in 2-week-old lactating *Cth*^−/−^ mice (7.43 versus 184 µM) although both levels were much higher than those in their respective WT mice (1.08 and 13.0 µM, respectively) [57]. This could be the reason for the “semi-” normal oxytocin signaling in the CNS (that regulates maternal behavior in dams and social memory/recognition in males) and the substantially impaired oxytocin signaling in the periphery (that regulates milk ejection/uterine contraction, especially the former in this study). Namely, high circulatory tHcy levels in *Cth*^−/−^ dams influenced on (high) tHcy in their whey (Table 1) but not circulatory tHcy in their pups (Table 2), and may not affect tHcy in their CNS significantly [57]. Whether hypertension/mild proteinuria in *Cth*^−/−^ pregnant mice could impair the peripheral oxytocin signaling or vice versa remains unknown. The molecular mechanisms underlying the deficits in *Cth*^−/−^ mice require further in vitro, ex vivo, and in vivo studies such as the detection of oxytocin-homocysteine dimers in the circulation or within the cells, and Otxr-downstream homocysteine target molecules within the cells.

In conclusion, this study revealed preeclampsia-like features and partial lactation failure in cystathioninuria model mice. Cth-deficient cystathioninuria patients are currently buried because they are considered free of apparent clinical manifestations and, unlike Cbs-deficient homocysteinemia patients, they are not detected in the current newborn screening that detects hypermethioninemia [21]. As lactation failure can be managed in clinical settings, this study may help the understanding of the pathophysiology of preeclampsia in relationship to homocysteine and transsulfuration pathway mediated by CBS and CTH.

## 4. Materials and Methods

### 4.1. Animals

*Cth*^+/−^ mice were generated and backcrossed for 10 generations to C57BL/6J inbred strain (CLEA Japan, Tokyo, Japan) [21]. The N10 (backcrossed 10 generations) *Cth*^+/−^ males and females were bred to obtain *Cth*^−/−^ mice. Mice were housed in an air-conditioned room (23 ± 1 °C, 55 ± 5% humidity), kept in a 12-h dark/light cycle, and allowed free access to a CE-2 standard dry rodent diet (CLEA Japan) and water. Before the surgeries, mice were anaesthetized with isoflurane. All animal procedures conformed to the Guide for the Care and Use of Laboratory Animals, 8th Edition published by the US National Research Council and were approved by the Animal Care Committees of Showa Pharmaceutical University (No. P-2016-10 and P-2018-07; approval dates: 22 July 2016 and 12 April 2018, respectively).

### 4.2. Blood Pressure/Heart Rate Measurement

Blood pressure was measured using a BP-98A tail-cuff manometer (Softron, Tokyo, Japan) [21,28]. Briefly, mice were settled with a 37 °C heated holder, and a balloon sensor surrounding the root of the tail was used to detect both the blood pressures and heart rates under no anesthesia.

### 4.3. Measurement of the Biochemical Parameters in the Serum and Urine

Blood and urine were collected from isoflurane-anaesthetized mice through cardiac and urinary bladder punctures, respectively. Serum (or urinary) levels of total protein, creatinine, urea nitrogen, AST, and ALT were measured using commercial colorimetric assay kits from Fujifilm-Wako (Osaka, Japan). For the blood oxytocin measurements, blood was collected from the retro-orbital plexus. Serum was prepared and its protein components were denatured by 0.05% trifluoroacetic acid and removed by the MonoSpin C18 column (GL Science, Tokyo, Japan) with acetonitrile elution. After acetonitrile evaporation, oxytocin contents were measured using the Oxytocin ELISA Kit (Enzo Life Sciences, CA, NY, USA) according to the manufacturer’s instruction.

### 4.4. Maternal Behavior Analyses

For examining the nesting activity score, postpartum mice were monitored using an infrared camera, and pictures were taken every 10 min before the birth and until 6 h after birth, or during 6–12 h after birth. When the dam crouched over more than half of her pups in one place, the dam was judged to have a nest. When the dam had a nest in 0–9, 10–18, 19–27, and 28–36 shots among a total of 36 shots, the nesting activity scores were evaluated as 0, 1, 2, and 3, respectively. For the retrieving activity, the latency to retrieve 1–4 pups after 5 pups were placed in the diagonal position of a rectangle cage (225 mm × 338 mm × 140 mm high) was examined. When more than half of the pups headed to their dam under her arms or belly in 0–9, 10–18, 19–27, and 28–36 shots among the total 36 shots, the breastfeeding activity scores were evaluated as 0, 1, 2, and 3, respectively. Namely, the breastfeeding activity score becomes higher when the dam has likely more chance to breastfeed their pups. The actual confirmation of breastfeeding was not executed not to stimulate lactating dams.

### 4.5. Histological Analyses of Mammary Glands

Mammary glands were isolated from lactating dams after isoflurane anesthetization. For whole-mount Carmine alum staining [58], isolated mammary glands were loaded on glass slides and fixed with Carnoy solution (ethanol:chloroform:acetic acid = 60:30:10) for 6 h at room temperature. The slides were washed with 70% ethanol and then double distilled water, immersed in Carmine alum solution (0.2% Carmine/0.5% aluminum potassium sulfate), and dehydrated/penetrated with ethanol and xylene. For hematoxylin/eosin staining, the isolated mammary glands were fixed in 10% neutral buffered formalin for 24 h and embedded in paraffin with Leica ASP200S. Then the paraffin-embedded blocks were prepared using Leica EG1160. The blocks were cut at 3 µm using Leica RM2265; the cuts were attached to MAS coated slide glasses (Matsunami Glass, Osaka, Japan), deparaffinized with xylene/ethanol, stained with hematoxylin/eosin solutions (Muto Pure Chemicals, Tokyo, Japan), and analyzed under the Keyence BZ-X800 microscope. The section pictures of the mammary lobules were analyzed using Adobe Photoshop (version 6).

For Cth immunostaining, after the deparaffinization with xylene/ethanol, the samples (on the slide glasses) were immersed in Immunosaver (Nisshin EM, Tokyo, Japan) at 98 °C for 45 min for antigen activation and blocked with 10% normal donkey serum, 1% casein, and 0.1% Tween 20 in phosphate buffered saline (PBS). The samples were incubated with anti-CTH rabbit polyclonal antibody [21,59] (1:100 dilution in Can Get Signal immunostain Solution A [Toyobo, Osaka, Japan]), and then with anti-rabbit IgG donkey polyclonal antibody conjugated with Alexa 594 (1:500 in Can Get Signal immunostain Solution A) and DAPI. Immunofluorescence was analyzed under the BZ-X800 microscope.

### 4.6. Western Blot Analyses

Each tissue aliquot (~100 mg) was homogenized in a lysis buffer (50 mM Tris-HCl (pH7.4), 150 mM NaCl, 1 mM EDTA, 1% (*w*/*v*) Triton X-100, 0.1% (*w/v*) phenylmethylsulfonyl fluoride, and Complete EDTA-free Protease Inhibitor Cocktail (Roche Diagnostics, Tokyo, Japan)) using a MicroSmash-100R (MS-100R) homogenizing system (Tomy, Tokyo, Japan) and zirconia beads (4100 rpm, 30 s × 3, 4 °C). Homogenates were centrifuged at 10,000× *g* for 10 min at 4 °C, and the supernatants were recentrifuged in the same way. After heating (100 °C, 3 min) with the sample buffer, samples (20 µg/lane) were resolved on 10% pre-cast PAGE gels, and then transferred to the Immobilon PVDF membrane (Millipore) and subjected to western blotting. Cth was detected with anti-CTH mouse monoclonal antibody (clone 4E1-1B7 from Abnova (Taipei, Taiwan); 1:5000 in Can Get Signal immunostain Solution 1 (Toyobo)) and mouse IgGκ BP-HRP (mouse IgGκ light chain binding protein-horseradish peroxidase from Santa Cruz; 1:10,000 in Tris buffered saline (TBS)-Tween 20). Oxr was detected with anti-Oxr rabbit monoclonal antibody (clone EPR12789 from Abcam (Cambridge, UK); 1:5000 dilution in Can Get Signal immunostain Solution 1) and anti-rabbit IgG, HRP-linked whole Ab donkey antibody (GE Healthcare; 1:10,000 in TBS-Tween 20). Glyceraldehyde 3-phosphate dehydrogenase (GAPDH) was detected with anti-GAPDH rabbit monoclonal antibody (clone 14C10 from Cell Signaling Technology (Danvers, MA, USA); 1:10,000 in TBS-Tween 20) as a loading control. Chemiluminescence detection was performed using Chemi-Lumi One Ultra (Nacalai Tesque, Kyoto, Japan) and the ATTO WSE-6100 LuminoGraph I imager (ATTO, Tokyo, Japan).

### 4.7. Milk Ejection Assay

The milk ejection assay was performed as described previously [60]. Lactating dams (whose pups were removed before 1 h) were anaesthetized with isoflurane and their breast mammary glands were exposed. First, 10 µL PBS (vehicle) was applied and a picture was taken after 1 min. Secondly, 0.04 unit oxytocin (in 10 µL PBS) was applied and the next picture was taken after 1 min. The presence and absence of the oxytocin response were scored as 1 and 0, respectively.

### 4.8. Uterine Contraction Assay

Adult female mice (8–10-week-old) were subcutaneously injected with 10 µg of 17β-estradiol (in 100 µL of 50% DMSO/50% PBS) on their backs and, after 2 days, uteruses on both sides were dissected out from anaesthetized mice. The uterus (~1.5 cm long) was mounted between two steel hooks in an isolated tissue chamber of the conventional Magnus apparatus containing artificial extracellular fluids (AEF; 148 mM NaCl, 4 mM KCl, 1.2 mM CaCl_2_, 1.5 mM MgCl_2_, 5 mM glucose, and 10 mM HEPES-Na (pH7.4)) at 37 °C. After 5 min of holding, methionine (final 2 mM), homocystine (final 200 µM), or vehicle (AEF) was applied to the chamber. After 30 min of incubation, oxytocin (final 0.1 unit/mL) was applied. After 5 min of recoding the contraction frequency, the chamber was washed once with AEF and filled with AEF containing 25 mM KCl to obtain the maximal contraction. The contraction (/relaxation) frequency per min and average peak heights (magnitudes) relative to those of 25 mM KCl were recorded.

### 4.9. Social Memory/Recognition Test

Social memory/recognition test was performed as described previously [37]. Both 8–10-week-old WT and *Cth*^−/−^ male mice were transferred from group to individual housing for a week before the test to permit the establishment of a home-cage territory. The test began when a stimulus WT female (8–10-week-old) was placed in the home cage of each male mice (*n* = 5 each) for a 1-min confrontation. Then the stimulus female was removed to an individual cage for 10 min. We repeated this sequence on the same male and female for four trials with 10-min intervals. In the 5th dishabituation trial, we introduced a new unfamiliar female. Behavior was video-recorded and the duration (s) spent for anogenital inspection was counted during each 1-min confrontation.

### 4.10. Amino acid Measurement of Serum and Whey Samples

Lactating dams (whose pups were removed before 2 h) were subcutaneously injected with oxytocin (4 units) and breast milk was collected by nipple massaging. The breast milk was centrifuged at 2300× *g* for 10 min at 4 °C to remove the fat contents, and then at 150,000× *g* for 10 min at 4 °C to collect the whey samples. The serum and whey samples were analyzed for their amino acid concentrations using amino acid/thiol-derivatization reagents as previously described in detail [61].

### 4.11. Statistical Analyses

Data were expressed as mean ± SD (*n*: sample numbers). Statistical comparison was performed using the two-tailed unpaired Student’s *t*-test, Mann–Whitney *U* test (only in Figure 3E,G), or Kaplan-Meier survival analyses using a Prizm 5 software (GraphPad, San Diego, CA, USA); all *p* values less than 0.05 denoted a significant difference.

## Figures and Tables

**Table 1:** Amino acid levels in serum and whey from virgin and primipara (with 2-week-old pups) of wild-type and *Cth*^−/−^ mice.

	Serum from Virgin		Serum from Primipara		Whey from Primiparas	
	WT (*n* = 9)	*Cth^−/−^* (*n* = 7)	WT (*n* = 7)	*Cth^−/−^* (*n* = 12)	WT (*n* = 7)	*Cth^−/−^* (*n* = 12)
Ala	446 ± 78	471 ± 115	618 ± 395	1164 ± 799 ^#^	309 ± 79	540 ± 121 ***
Arg	133 ± 27	196 ± 39 **	46.0 ± 40.3 ^##^	25.8 ± 27.1 ^##^	35.3 ± 19.3	31.8 ± 9.7
Asp/Asn	12.4 ± 5.7	15.0 ± 9.9	16.6 ± 5.0	15.2 ± 6.9	40.8 ± 15.8	24.5 ± 8.0 *
Gln	837 ± 125	846 ± 144	873 ± 318	1344 ± 482 *^,##^	74.4 ± 21.1	181 ± 46 ***
Glu	20.7 ± 11.8	18.8 ± 11.6	58.5 ± 30.2 ^#^	36.2 ± 19.2 ^#^	211 ± 48	190 ± 43
Gly	403 ± 60	424 ± 71	359 ± 153	520 ± 185	271 ± 60	414 ± 77 ***
His	41.6 ± 7.4	85.3 ± 11.5 ***	51.9 ± 25.4	195 ± 67 ***^,##^	7.07 ± 2.52	24.0 ± 5.0 ***
Ile	121 ± 16	110 ± 33	113 ± 44	169 ± 60 *^,#^	29.7 ± 19.4	35.0 ± 14.8
Leu	156 ± 13	153 ± 45	158 ± 77	274 ± 113 *^,##^	24.6 ± 8.9	34.9 ± 13.6
Lys	277 ± 50	287 ± 87	290 ± 89	663 ± 292 **^,##^	70.0 ± 19.2	113 ± 37 **
Met	72.0 ± 8.8	94.2 ± 10.8 ***	128 ± 51^#^	147 ± 44 ^##^	5.49 ± 1.22	10.1 ± 2.7 ***
Phe	96.5 ± 17.6	89.1 ± 15.9	93.2 ± 53.3	92.4 ± 39.9	1.25 ± 1.34	2.60 ± 1.25
Pro	121 ± 20	140 ± 50	298 ± 237	288 ± 187 ^#^	312 ± 177	237 ± 71
Ser	166 ± 34	162 ± 37	189 ± 98	239 ± 96 ^#^	234 ± 66	306 ± 84
Thr	185 ± 28	190 ± 60	248 ± 108	368 ± 148 ^##^	111 ± 38	158 ± 40 *
Tyr	156 ± 29	148 ± 45	86.8 ± 61.5 ^#^	145 ± 86	N.D.	N.D.
Val	258 ± 21	239 ± 80	234 ± 87	386 ± 165 *^,#^	76.0 ± 43.5	105 ± 59
Cystathionine	18.5 ± 3.0	127 ± 34 ***	54.7 ± 44.0	606 ± 298 ***^,##^	36.9 ± 14.8	361 ± 95 ***
Citrulline	85.3 ± 8.9	215 ± 57 ***	65.8 ± 14.4 ^#^	242 ± 77 ***	6.76 ± 0.88	32.3 ± 8.0 ***
Ornithine	68.1 ± 9.9	87.1 ± 25.1	92.2 ± 47.1	220 ± 131 **^,##^	21.5 ± 4.0	58.9 ± 14.4 ***
Taurine	385 ± 247	487 ± 226	835 ± 256 ^##^	725 ± 422	384 ± 27	396 ± 89
Total Cys	289 ± 41	197 ± 18 ***	508 ± 82 ^##^	384 ± 85 **^,##^	154 ± 80	164 ± 35
Total Hcy	10.8 ± 5.8	181 ± 16 ***	11.3 ± 1.8	111 ± 24 ***^,##^	6.09 ± 11.33	49.6 ± 13.4 ***
Total GSH	97.5 ± 52.1	68.4 ± 16.6	176 ± 76 ^#^	136 ± 120	23.6 ± 3.5	22.3 ± 3.7
Total Cys-gly	3.71 ± 0.7	N.D.	5.93 ± 1.95 ^#^	N.D.	41.7 ± 8.4	43.2 ± 14.8
Total γ-Glu-Cys	11.2 ± 1.6	7.32 ± 0.31 **	21.1 ± 4.9 ^##^	12.2 ± 3.3 **^,##^	27.3 ± 48.1	43.3 ± 19.0

Mean ± SD from independent mouse samples are presented. N.D., not detected. The differences versus wild-type (WT) samples were significant at * *p* < 0.05, ** *p* < 0.01, and *** *p* < 0.001, and those versus virgin samples were at ^#^ *p* < 0.05 and ^##^ *p* < 0.01 in Student *t*-test.

**Table 2:** Serum amino acid levels at 6 and 12 h after birth in *Cth*^+/−^ pups born to wild-type or *Cth*^−/−^ dam mice.

	*Cth*^+/−^ Pups at 6 h after Birth		*Cth*^+/−^ Pups at 12 h after Birth	
	From Wild-Type	From *Cth*^−/−^	From Wild-Type	From *Cth*^−/−^
	(*n* = 3 Litters)	(*n* = 5 Litters)	(*n* = 4 Litters)	(*n* = 4 Litters)
Ala	449 ± 392	219 ± 34	163 ± 23	168 ± 27 ^#^
Arg	6.72 ± 0.13	18.0 ± 13.1	33.0 ± 18.2	26.8 ± 9.8
Asp/Asn	115 ± 44	104 ± 22	96.4 ± 25.4	122 ± 22
Gln	1801 ± 888	1666 ± 284	1585 ± 408	1661 ± 285
Glu	104 ± 28	92.2 ± 28.8	86.0 ± 10.2	94.6 ± 2
Gly	240 ± 46	176 ± 32	209 ± 24	216 ± 36
His	20.6 ± 2.1	20.2 ± 7.6	15.2 ± 0.7 ^#^	17.3 ± 4.3
Ile	83.2 ± 35.8	70.0 ± 14.5	79.3 ± 5.6	82.2 ± 23
Leu	137 ± 60	110 ± 26	110 ± 5	114 ± 36
Lys	752 ± 194	625 ± 94	580 ± 47	668 ± 100
Met	118 ± 23	136 ± 26	83.6 ± 3.2	96.3 ± 16.3 ^#^
Phe	118 ± 34	117 ± 21	112 ± 6	120 ± 16
Pro	188 ± 107	155 ± 42	174 ± 9	171 ± 35
Ser	57.3 ± 11.6	57.2 ± 9.1	71.4 ± 7.8	62.6 ± 12.3
Thr	197 ± 102	170 ± 34	122 ± 9	127 ± 23
Tyr	140 ± 57	138 ± 31	105 ± 10	120 ± 22
Val	218 ± 64	180 ± 21	148 ± 7	160 ± 37
Cystathionine	97.5 ± 15.7	110 ± 21	80.8 ± 16.8	100 ± 10
Citrulline	56.6 ± 7	66.7 ± 10.9	64.1 ± 3.2	68.0 ± 6.7
Ornithine	131 ± 42	121 ± 15	96.9 ± 22.5	113 ± 35
Taurine	1874 ± 489	1334 ± 172	1292 ± 204	1455 ± 201
Total Cys	271 ± 15	281 ± 37	259 ± 16	279 ± 34
Total Hcy	4.93 ± 0.50	10.7 ± 3.7 *	3.98 ± 0.92	6.41 ± 2.63
Total GSH	105 ± 46	68.2 ± 26.0	67.7 ± 13.7	69.2 ± 12.3
Total γ-Glu-Cys	20.0 ± 6.6	23.4 ± 2.1	24.9 ± 4.6	23.6 ± 3.0

Mean ± SD from independent mouse samples are presented. N.D., not detected. The differences versus *Cth*^+/−^ pups born from wild-type dams were significant at * *p* < 0.05, and those versus *Cth*^+/−^ pups at 6 h after birth born from the respective dams at ^#^ *p* < 0.05.

## Abbreviations

AEF	artificial extracellular fluid
Cbs	cystathionine β-synthase
CNS	central nervous system
Cth	cystathionine γ-lyase
CVD	cardiovascular disease
H_2_S	Hydrogen sulfide
NTD	neural tube defect
Oxtr	oxytocin receptor
PPG	propargylglycine
V2r	vasopressin receptor 2
WT	wild-type
